# Challenge for Affective Brain-Computer Interfaces: Non-stationary Spatio-spectral EEG Oscillations of Emotional Responses

**DOI:** 10.3389/fnhum.2019.00366

**Published:** 2019-10-30

**Authors:** Yi-Wei Shen, Yuan-Pin Lin

**Affiliations:** Institute of Medical Science and Technology, National Sun Yat-sen University, Kaohsiung, Taiwan

**Keywords:** affective brain-computer interface, EEG, intra-individual difference, inter-individual difference, independent component analysis

## Abstract

Electroencephalogram (EEG)-based affective brain-computer interfaces (aBCIs) have been attracting ever-growing interest and research resources. Whereas most previous neuroscience studies have focused on single-day/-session recording and sensor-level analysis, less effort has been invested in assessing the fundamental nature of non-stationary EEG oscillations underlying emotional responses across days and individuals. This work thus aimed to use a data-driven blind source separation method, i.e., independent component analysis (ICA), to derive emotion-relevant spatio-spectral EEG source oscillations and assess the extent of non-stationarity. To this end, this work conducted an 8-day music-listening experiment (i.e., roughly interspaced over 2 months) and recorded whole-scalp 30-ch EEG data from 10 subjects. Given the large size of the data (i.e., from 80 sessions), results indicated that EEG non-stationarity was clearly revealed in the numbers and locations of brain sources of interest as well as their spectral modulation to the emotional responses. Less than half of subjects (two to four) showed the same relatively day-stationary (source reproducibility >6 days) spatio-spectral tendency towards one of the binary valence and arousal states. This work substantially advances the previous work by exploiting intra- and inter-individual EEG variability in an ecological multiday scenario. Such EEG non-stationarity may inevitably present a great challenge for the development of an accurate, robust, and generalized emotion-classification model.

## Introduction

Electroencephalogram (EEG)-based affective brain-computer interfaces (aBCIs) have been attracting ever-growing interest and research resources. The aBCI represents an external device with a capacity for emotional awareness based on its interaction with a user’s emotional responses. Recent availability of user-friendly wearable EEG sensing technologies and their market profitably bring laboratory-oriented aBCI research closer to practical applications in multidisciplinary domains such as NeuroMarketing, NeuroRehabilitation, and NeuroGaming. To this end, an embedded framework in aBCI that enables the accurate and reliable recognition of emotional states of interest from time-varying, spatio-spectral EEG oscillations is of tremendous interest. Considerable work has been carried out to develop a machine learning framework to this end. The leveraged framework typically combines signal processing, feature engineering, and feature classification (Lin et al., 2010b; Jenke et al., 2014; Zheng, 2017; Xing et al., 2019). This research in machine-learning has rapidly progressed and contributed to our understanding of EEG oscillation modeling underlying emotional responses in general.

Nonetheless, the brain often switches between different operational modes while engaging in a task in realistic environments (Lance et al., 2015). This may be attributed to changes in several behavioral and/or psychophysiological states such as attention, stress, anxiety, or sleep quality. For an individual, the task-relevant EEG oscillations of interest may change on a daily basis, especially in emotional perception and experience. The EEG patterns are thus likely to be different on different days, considered to be reflective of inter-day non-stationarity or intra-individual variability. Some work has focused on empirically assessing such day-to-day variability and its negative impact on machine-learning proficiency in affective computing (Chai et al., 2017; Lin et al., 2017; Liu et al., 2018). In other words, the same emotion across days tended to be more widely scattered than the data clusters of different emotions within the same day (Lin et al., 2015). Such inter-day non-stationarity inevitably makes emotion prediction by a pre-trained emotion-aware model more difficult given the discrepancy between EEG distributions from different days. Until now, recent endeavors have focused on integrating advanced signal processing techniques or additional data calibration (Chai et al., 2017; Lin et al., 2017; Liu et al., 2018) to tackle this intra-individual variability, though the proposed scenario or the corresponding improvements still remain limited in their ability to perform robust predictions.

In addition, substantial non-stationary EEG correlates of emotional responses also exist between individuals, reflective of, namely, inter-individual non-stationarity or inter-individual variability. Due to intrinsic differences in personality, culture, gender, educational background, and/or living environment, individuals may have distinct behavioral and/or neurophysiological responses even while perceiving the same event. They are thus not likely to share common EEG distributions corresponding to the same emotional states, meaning that the performance of a generic machine-learning model will either be compromised or fail for certain individuals. Some related work has explored the negative impact of inter-individual non-stationarity on affective computing (Lin et al., 2010b; Soleymani et al., 2012; Lin and Jung, 2017; Li et al., 2019; Xing et al., 2019). In other words, a subject-independent model (i.e., in which learning has been carried out on the aggregated data of all available individuals) did not exclusively outperform a subject-dependent counterpart due to the increased amount of training data. Taken together, EEG non-stationarity (intra- and inter-individual counterparts) represents a great challenge to the development of an accurate, robust, and generalized emotion-classification model, and thereby considerately hinders the practical applicability of an aBCI to a realistic environment.

While most work has searched for better cross-day or cross-individual prediction by means of novel signal processing and machine learning frameworks, less effort has been directed at pinpointing the fundamental nature of the non-stationary EEG oscillations underlying emotional responses across both days and individuals. This work is thus devoted to using a data-driven blind source separation method, i.e., independent component analysis (ICA), to exploit emotion-relevant spatio-spectral EEG source oscillations and assess the extent of the non-stationarity in terms of the spatial configuration of cortical sources and the statistical properties of their tempo-spectral activities. To this end, this work conducted an 8-day music-listening experiment (i.e., roughly interspaced over the course of 2 months) and recorded whole-scalp 30-ch EEG data from a group of 10 subjects. This big dataset (80 sessions) allowed us to systematically investigate intra- and inter-individual EEG non-stationarity through source-level analysis. The empirical outcomes of this work not only advance previous work in EEG neuroscience that focused on single-day/-session recordings (Lin et al., 2010a; Rogenmoser et al., 2016) and sensor-level analysis (Schmidt and Trainor, 2001; Sammler et al., 2007; Daly et al., 2014), but also empirically demonstrate how challenging it is to deploy a robust emotion-aware analytical infrastructure given ecological EEG non-stationarity.

## Materials and Methods

### Participants

Ten healthy subjects (six males, four females; age 23.3 ± 0.82 years) participated in an 8-day music-listening experiment interspaced over the course of 2 months (approximately once per week with an average time interval of 7.94 ± 1.76 days). All subjects were undergraduate or graduate students in the College of Engineering or Science. They had not received professional training on musicology or musical instruments and were thus considered to be non-musicians. They read and signed a consent form prior to the longitudinal experiment, which was approved by Human Research Protections Program of the local ethics committee. All subjects completed the entire eight-session experiment even though they were allowed to voluntarily withdraw at any time. The experiment facilitated an EEG analysis of emotional responses from a total of 80 day-sessions.

### Experimental Design and Procedure

Prior to the music-listening experiment, all subjects were asked to provide a list of their favorite songs which, in their daily life, are able to emotionally arouse them. They were instructed, following the 2D valence-arousal emotion model (Russell, 1980), to select five songs from each of the four emotion quadrants (i.e., positive valence–high arousal, negative valence–high arousal, negative valence–low arousal, and positive valence–low arousal states) and further extract from each song a 60-s excerpt for use in the EEG-recording experiment. In order to avoid startling effects, the beginning and end of each excerpt were each faded in and out over the course of 10 s. The finalized 60-s song highlights were confirmed by each subject prior to the experiment. In addition, along with a self-selected set per subject, this study randomly recruited extra four excerpts (one per quadrant) from other subjects to form his/her music procedure. This was intended to explore the relationship between familiarity and emotional responses in a longitudinal experiment, though this was not the analytical focus of this study.

The 24 song excerpts from each subject were separated into six four-trial blocks. Each block contained an excerpt for each emotional quadrant in random order. Each trial began with a 30-s resting phase followed by a 60-s music listening phase and ended with a self-reported rating task. In the rating task, subjects were required to rate songs on a five-point scale of emotional valence (from negative to positive), emotional arousal (from calm to excited), preference (from dislike to like), and familiarity (from never heard to knew well) based on what they had felt on each day. They did not necessarily assign the same scores as those assigned previously or as those provided in the selection of the songs. The experimental protocol was entirely self-paced such that each subject decided the amount of rest time before proceeding to the next trial or block. The music-listening experiment took place in a dimly lit room. The subjects were instructed to remain seated, keep their eyes closed (an auditory cue for every self-rating task), minimize their body movements, and fully attend to the song excerpts played through speakers during the entire experiment. Each subject listened to his/her unique set of 24 60-s songs in a shuffled order on each of the 8 days.

### EEG Acquisition

EEG signals were recorded using a 36-channel EEG system (Neuroscan, Compumedics Ltd., Abbotsford, VIC, Australia). The 30 scalp electrodes were placed according to the International 10–20 system, with the linked mastoids (average of A1 and A2) and forehead as reference and ground sites, respectively. Four auxiliary electrodes were also placed to monitor electrooculogram (EOG) activity (two for above and below the left eye and another two on the outer canthi). All electrode impedance values were kept below 15 kΩ for better signal quality. EEG signals were sampled at 500 Hz and in a bandwidth of 1–100 Hz with a 60 Hz notch filter to remove powerline contamination.

### Exploring Stationary Spatio-spectral EEG Oscillations

The adopted analytical framework included a number of steps to explore stationary spatio-spectral EEG oscillations of emotional responses for the 8-day dataset of each subject, including artifact suppression, ICA and clustering, and statistical assessment of emotional valence and arousal states. Data analysis and visualization were performed using the open source EEGLab toolbox/scripts (Delorme and Makeig, 2004) and MATLAB functions/scripts (The Mathworks, Inc., Natick, MA, USA). Details of technical procedures and implementation are provided below.

EEG data of each single-day session were band-passed filtered to 1–50 Hz to suppress low-frequency drifts and high-frequency artifacts. Artifact subspace reconstruction (ASR; Kothe and Jung, 2015) was then used to compensate for high-variance artifacts from the filtered EEG signals (Mullen et al., 2015; Artoni et al., 2017; the user-defined threshold was set to 5 standard deviations in this study), followed by a visual inspection to ensure data quality prior to the subsequent ICA analysis. Given the available 80 sessions (10 subjects × 8 sessions), only ~1% on average (within a range of 0.1%–7%) of data from a single-day session was removed prior to further analysis.

The preprocessed single-day EEG data was submitted to ICA separately to parse the multichannel signals into independent components (ICs) via an extended infomax ICA algorithm. To localize the sources of the decomposed ICs, a single-dipole source model best fitted to the IC’s scalp projection was calculated using a boundary element head model (BEM) based on the MNI brain template (Montreal Neurological Institute, MNI, Montreal, QC, Canada) implemented using the DIPFIT routine (Oostenveld and Oostendorp, 2002). Among the 30 derived ICs (four EOG and two reference channels excluded), this study evaluated scalp maps, spectral profiles, single dipole-fitting efficiency (explaining >85% of variance of the IC scalp map, as in Onton and Makeig, 2006), and within-brain dipole locations to retain cortical brain sources yet discard stereotyped non-cortical artifactual counterparts (e.g., eye movements, sporadic muscle tensions) prior to further analysis. The above ICA procedures and screening criteria are commonly used in other studies (Delorme et al., 2012; Wagner et al., 2016). On average, 88.50 ± 16.28 cortical ICs were retained in each subject in the 8-day dataset (11.20 ± 1.96 ICs per single-day session). Next, to assess stationary spatio-spectral sources across days, a *K*-means clustering algorithm was used to categorize similar ICs across 8 days into distinct IC clusters for each individual based on the attributes of their power spectral densities, scalp maps, and 3D dipole locations. ICs with distance values more than 3 standard deviations from the mean of their cluster centroids were relocated to another suitable one or classified as outliers. Such a semi-automatic IC clustering procedure allowed for the aggregation of neurophysiologically interpretable brain sources featuring homogeneous scalp maps and spectral profiles, thereby facilitating the assessment of their stationarity over the course of multiple days. This work adopted two objective measurements, namely dipolarity (Delorme et al., 2012) and reproducibility, to quantify both how well dipolar brain sources of interest were exploited on each single day and how frequently they emerged across days. The dipolarity value in this study represented the percentage of data variance accounting for a single dipole-fitting of the IC scalp map. The higher the dipolarity value, the more dipolar and prone to neurophysiological assessment of the brain source. Reproducibility was intuitively defined as the percentage of day sessions yielding the same dipolar ICs. In other words, a dipolar IC with 100% reproducibility means it is present in each of the 8 days.

In order to further assess spectral correlations between the derived ICs and emotional responses in distinct frequency bands, the short-time Fourier transform with a 50% overlapped 2-s Hamming window was used to estimate their spectrograms. The spectra were then grouped into five typical bands, namely delta (1–3 Hz), theta (4–7 Hz), alpha (8–13 Hz), beta (14–30 Hz), and gamma (31–50 Hz) bands. Each logarithmic band-power time series belonging to a 60-s music excerpt was normalized by subtracting the mean power and dividing by the standard deviation of its preceding 30-s resting phase, followed by the single-trial baseline normalization manner in Grandchamp and Delorme (2011).

### Statistical Assessment of Spatio-spectral Oscillations vs. Emotional States

This work attempted to exploit stationary spatio-spectral EEG correlates of emotional responses. All neurophysiological and behavioral responses regarding self-reported valence and arousal ratings were evaluated across multiple days for each individual. To this end, the 60-s band-specific spectral time series of the grouped ICs were categorized according to their assigned dichotomized valence (positive vs. negative) and arousal (high vs. low) states and assessed for whether there was a set of relatively day-stationary spatio-spectral oscillations modulated by emotional responses. The dichotomization was determined by setting a threshold at the middle of the five-point rating scale, i.e., <3 for negative valence/low arousal and >3 for positive valence/high arousal labels. This may have led to an imbalance in the classification of samples according to this binary classification scheme in each daily session. This work thus employed an unpaired *t*-test to assess the relationship across days between 8-day spatio-spectral EEG oscillations and emotional responses in each individual. For the daily association, a non-parametric permutation test was adopted since the limited 24 dichotomized trials per day may not comply with the assumption of a parametric approach. The permutation was done by iteratively shuffling the labels (*n* = 20,000) over trials and computing the test statistic, forming a distribution of test statistic values under the null hypothesis. Statistical assessment was then conducted by comparing the observed test statistic value (without shuffled data) against the distribution of null-hypothesis test statistic values. This work further stressed the behavior of emotion response-categorized EEG oscillations on each day as a reference.

## Results

### Behavioral Ratings

**Figure 1:**
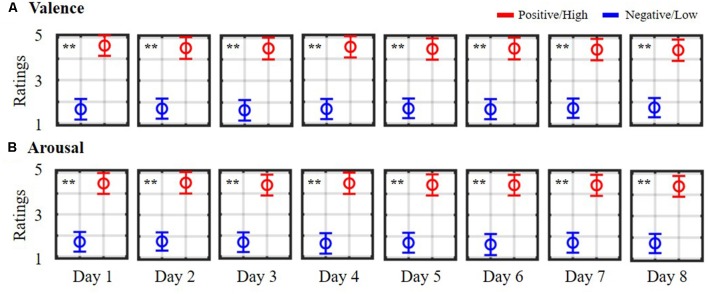
Daily self-reported ratings of emotional **(A)** valence and **(B)** arousal states. Ratings were summarized from 10 subjects participating in an 8-day music-listening experiment. The symbols are color-coded according to the adopted dichotomized threshold (= 3) in the five-point scale. Red symbols (>3) indicate positive valence/high arousal ratings, whereas blue symbols (<3) indicate negative valence/low arousal ratings. ** Indicates a statistical significance of *p* < 0.01.

Figure 1 depicts the daily self-reported ratings of 10 subjects while participating in the 8-day music-listening experiment. The dichotomization (=3) on the five-point scale of valence and arousal states led to averaged trials of 10.70 ± 0.25 vs. 11.30 ± 0.44 (positive vs. negative valence) and of 12.40 ± 0.47 vs. 9.40 ± 0.58 (high vs. low arousal) for each day session, and corresponded to a total of 85.70 ± 12.70 vs. 90.70 ± 18.57 and 114.50 ± 28.30 vs. 56.70 ± 34.35 for binary valence and arousal classes on average, accounting for eight sessions in each individual. However, the data of two subjects who happened to assign fewer labels with low arousal over the course of the eight sessions (<mean-standard deviation) were excluded from the arousal analysis. As can be seen, according to the unpaired *t*-test, the valence ratings differed significantly between positive and negative outcomes on each day session (*p* < 0.01). All eight sessions resulted in mean scores of 4.49 ± 0.08 and 1.69 ± 0.04 for positive and negative states, respectively. On the other hand, daily high arousal ratings were consistently higher than the low ones (*p* < 0.01), yielding 8-day mean ratings of 4.45 ± 0.05 and 1.68 ± 0.05 for high and low arousal states, respectively. The derived 8-day EEG trials and their self-reported binary labels from 10 subjects facilitated the subsequent exploratory assessment of (non)stationary spatio-spectral EEG dynamics of emotional responses.

### Demonstrating Day-Stationary Spatio-spectral EEG Oscillations and Their Associations With Emotional Responses From a Representative Subject

**Figure 2:**
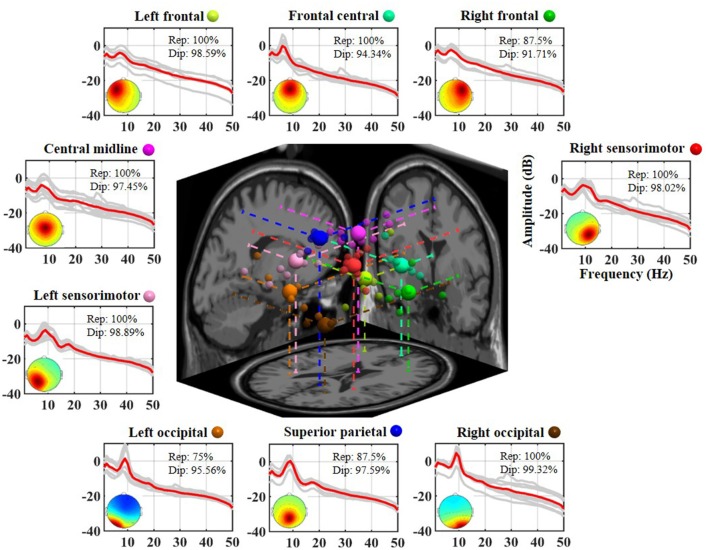
Eight-day cortical source reproducibility from a representative subject. Each surrounding subplot refers to an aggregated independent component (IC) cluster. Averaged and individual IC log-power spectra (dB) are plotted in red and gray lines, respectively, and the corresponding mean scalp maps of clusters are superimposed. Rep and Dip indicate reproducibility and mean dipolarity per IC cluster, respectively. The centered subplot represents a 3D overview of the equivalent dipole locations of the nine clusters and their projections onto the MNI brain template. Dots in the same color represent the ICs grouped into the same cluster, in which bigger dots represent the cluster centroids.

Figure 2 illustrates the neurophysiologically plausible IC clusters commonly exploited in the 8-day dataset from a representative subject. Nine ICs appeared to be relatively reproducible across days (reproducibility >75%, at least 6 of 8 days) and returned high estimated single-dipole brain sources (dipolarity >91%) that were spatially located in left frontal, frontal central, right frontal, left sensorimotor, central midline, right sensorimotor, left occipital, superior parietal, and right occipital brain regions. Each aggregated IC cluster corresponded to similar characteristics in terms of their logarithmic spectral profiles and 3D dipole source locations on the MNI brain template. Among them, the three frontal clusters and central midline cluster demonstrated a major peak in the theta band, and the others demonstrated a prominent alpha peak, in which the sensorimotor and superior parietal counterparts accompanied a minor beta peak.

**Figure 3:**
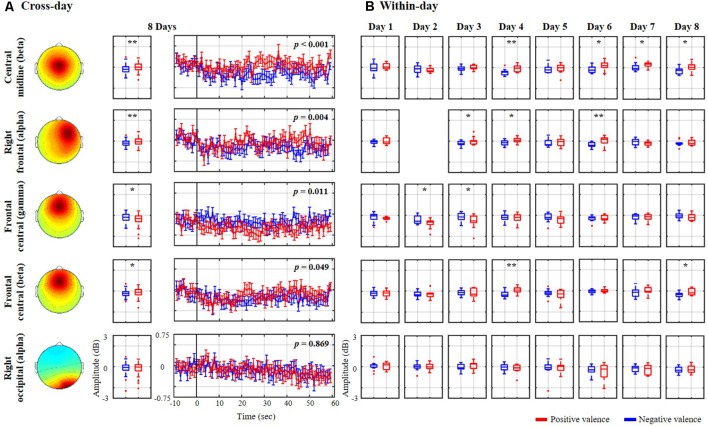
Valence-relevant spatio-spectral oscillations using **(A)** cross-day and **(B)** within-day analyses from the same representative subject shown in Figure 2. The cross-day analysis led to four meaningful spatio-spectral oscillations from the nine IC clusters. The insignificant right occipital alpha in the last row was also provided as a technical control. Red and blue profiles represent the spectral oscillations associated with a positive and negative valence, respectively. * and ** refer to a statistical significance of *p* < 0.05 and *p* < 0.01, respectively.

Figure 3 explores the association of the spectral oscillations of the nine exploited IC clusters with the binary valence states from the same representative subject (as shown in Figure 2). The cross-day outcome was summarized by leveraging the trials of all eight sessions together to benchmark the within-day counterpart. As shown in Figure 3A, the cross-day analysis demonstrated that four spatio-spectral oscillations were significantly altered according to positive vs. negative valence (*p* < 0.05), consisting of central midline beta, right frontal alpha, and frontal central beta and gamma bands. A valence-irrelevant outcome of right occipital alpha (*p* = 0.869) was also provided in the last row as a technical control. As can be seen, after the eyes-closed baseline, the state-wise spectral time series notably diverged from one another over the course of the 60-s excerpt. The negative valence led to a major drop in central midline beta and right frontal alpha (*p* < 0.01) and a marginal drop in frontal central beta (*p* = 0.049), whereas the positive valence accompanied a gamma decrease over the frontal central region (*p* < 0.05). Moreover, the cross-day analysis reflected a similar spectral tendency for most single days that were obtained by the within-day analysis. However, certain days happened to present reciprocal or distinctive outcomes. Taking the central midline beta as an example, 5 of 8 days (days 3, 4, 5, 6, and 8) exhibited a consistent decrease in negative compared to positive valence (days 4, 6, and 8 with *p* < 0.05), on day 2 there was a tendency towards a drop in positive valence, on day 7 positive valence tended to increase, and on day 1 spectral distinction was barely re-established. Such discrepancies in the cross- and within-day analysis more or less emerged in the other three informative spatio-spectral oscillations of interest from this representative subject. As the technical benchmark, the cross-day outcome of the right occipital alpha did reflect a common tendency towards indistinguishable spectral profiles on each individual day.

**Figure 4:**
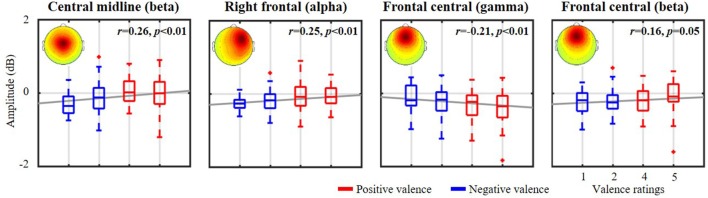
The relationship between valence-relevant spatio-spectral oscillations and self-reported ratings from the same representative subject shown in Figures 2, 3. The five-point scale of emotional valence was divided into two groups corresponding to scores below and above 3. Red and blue profiles represent positive (>3, more positive) and negative valences (<3, more negative), respectively. The gray lines depict the linear relationship as assessed by linear regression analysis. The mean scalp maps of the informative IC clusters are superimposed on each subplot.

Figure 4 further demonstrates the validity of the exploited emotional valence-relevant spatio-spectral oscillations on the initial five-point scale from the same representative subject, as shown in Figures 2, 3. With respect to the baseline, the spatio-spectral fluctuations of interest tended to be statistically modulated by the rating scale. Stronger negative ratings resulted in accentuated power attenuation in central midline beta (*r* = 0.26, *p* < 0.01), right frontal alpha (*r* = 0.25, *p* < 0.01), and frontal central beta (*r* = 0.16, *p* = 0.05) frequencies. In contrast, stronger positive ratings were linked to attenuated frontal central gamma deterioration (*r* = −0.21, *p* < 0.01). According to the empirical demonstration on the representative subject, this work as such applied the cross-day ICA analytical framework to each of the 10 subjects separately, exploring inter-subject commonality of the relatively day-stationary spatio-spectral EEG oscillations associated with emotional responses.

### Exploring the Inter-subject Commonality of the Day-Stationary Spatio-spectral EEG Oscillations and Their Associations With Emotional Responses

**Figure 5:**
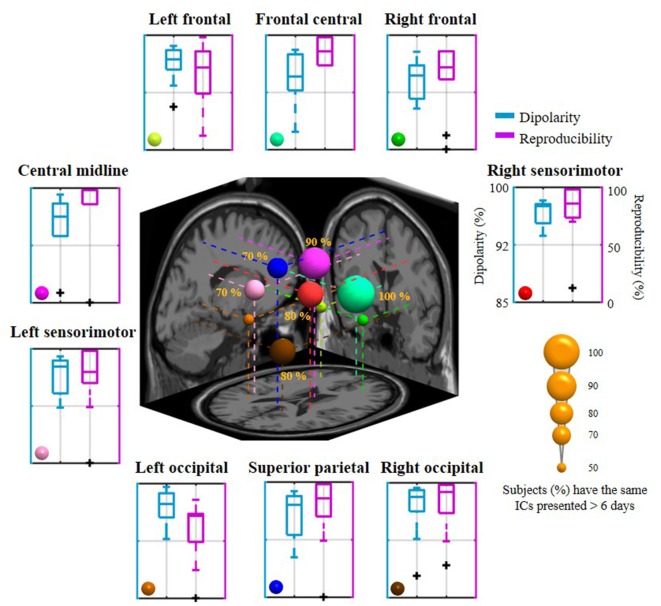
The mean dipolarity and reproducibility of the 8-day aggregated nine IC clusters for 10 subjects and their inter-subject commonality. Each surrounding subplot refers to the mean of an IC cluster summarized across subjects. The centered subplot represents a 3D overview of the dipole centroids of the nine clusters and their projections onto the MNI brain template, where the dipole size was scaled by the inter-subject commonality of the relatively day-stationary ICs (the percentage of subjects with consistently the same IC appeared in 6 of 8 days).

Figure 5 summarizes the dipolarity and reproducibility of the exploited 8-day aggregated nine IC clusters from 10 subjects. Each IC cluster yielded a mean dipolarity of >94.28%, and their grand mean dipolarity was 95.99 ± 1.04%, indicating their neurophysiological adequacy for the sequential spectral assessment of emotional responses. In contrast, mean reproducibility in the nine clusters varied from 60.89 ± 28.36% (in the left occipital cluster) to 88.57 ± 10.98% (in the frontal central cluster) and the grand mean reproducibility was 76.03 ± 10.40%. In the worst-case scenario, some of them happened to be completely absent from distinct subjects, such as in the right frontal, central midline, left sensorimotor, left occipital, and superior parietal sources (extreme outliers are shown in the boxplot). In order to evaluate the commonality of relatively day-stationary ICs for most subjects, this work defined a criterion by empirically counting ICs that were consistently present at least over the course of *N* days in the 8-day recording setting. *N* was set to six in this work due to the resultant nine-cluster mean reproducibility (i.e., 75% represents 6 of 8 days). Given the 6-day criterion, the inter-subject commonality (i.e., the percentage of the recruited 10 subjects with the same ICs over 6 days) was found to vary from 50% to 100%. The frontal central source was presented for each subject (100%), followed by the central midline source (90%), the right sensorimotor and the right occipital sources (80%), the left sensorimotor and the superior parietal sources (70%). The remaining three sources located in the left and right frontal regions and the left occipital regions had lower commonality (50%). The mean inter-subject commonality for the nine clusters was 71.11 ± 18.33% with cross-day reproducibility >6 days. The discernible cluster-to-cluster reproducibility and their inter-subject commonality reflected the non-stationarity of IC sources for each day and for each subject.

**Figure 6:**
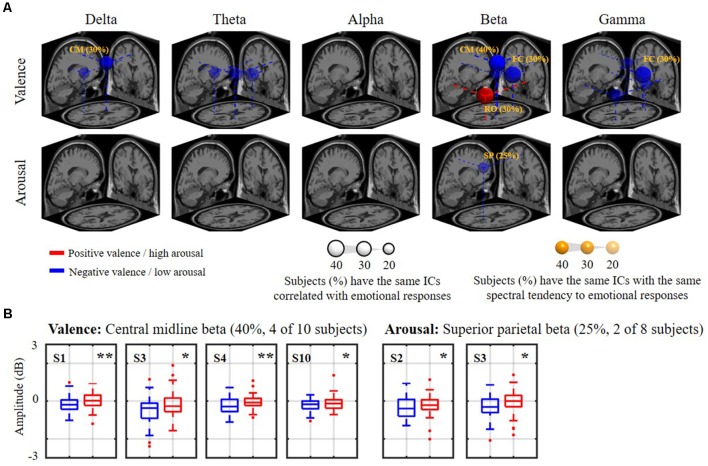
The relatively day-stationary, subject-common spatio-spectral oscillations in response to the binary valence and arousal states. Panel **(A)** refers to a 3D overview of emotion-relevant IC cluster centroids and their projections onto the MNI brain template (FC: frontal central, CM: central midline, RO: right occipital, SP: superior parietal). Sphere size was scaled to indicate the percentage of subjects with the same day-stationary IC significantly related to emotional responses (*p* < 0.05), and transparency further represents the percentage of subjects with the same spectral tendency towards an emotional label of the target ICs (annotated %). Only results above 20%, i.e., with at least two subjects in common, are shown. Red and blue colors represent the power suppression associated with positive valence/high arousal and negative valence/low arousal states, respectively. Panel **(B)** demonstrates valence and arousal outcomes with the highest inter-subject commonality. * and ** refer to a statistical significance of *p* < 0.05 and *p* < 0.01, respectively. Two subjects with highly imbalanced labels were excluded from the arousal analysis.

Figure 6 shows how the relatively day-stationary, subject-common ICs behaved in accordance with the emotional responses and whether they demonstrated the same spectral tendency towards the same binary state. Two main findings are mapped onto the MNI brain template in Figure 6A, including the percentage of subjects with the same ICs whose spectral oscillation statistically differed between the two binary states (*p* < 0.05) and the percentage of subjects with the same spatio-spectral tendency towards one of the two binary states (*p* < 0.05). The use of a large and more solid dipole means that analogous day-stationary spatio-spectral EEG correlates of an emotional state can be seen across more subjects. The Talairach coordinates of the centroids of the dipole distribution for each IC cluster and the relatively stationary outcomes for each emotion category are represented in Table 1. In general, the valence category yielded a higher inter-subject commonality for the spatio-spectral association across days. Four of 10 subjects similarly possessed central midline beta oscillations that significantly differed between the two binary states (i.e., the same emotion-related IC: 40%). They further led to more beta suppression for the negative valance compared to the positive counterpart (i.e., spatio-spectral tendency: 40%), as shown in Figure 6B. The negative valence also manifested the suppression in central midline delta power and frontal central beta and gamma power (i.e., spatio-spectral tendency: 30%). In addition, the positive valence tended to be associated with more right occipital beta suppression (30%). Other spatio-spectral tendencies typically had less commonality (20%). Unlike the valence outcome, the arousal category had worse inter-subject commonality. Only two of eight subjects (25%) were found to have similar superior parietal beta suppression in low arousal compared to high counterpart. Other spatio-spectral oscillations behaved quite inconsistently across individuals (<20%, with no consensus found between two subjects).

**Table 1:** Major day-stationary independent component (IC) clusters and their inter-subject commonalty to the binary valence and arousal states.

Emotional category	Source	Talairach coordinates			BA	Band power	Subjects (%) with the same emotion-related IC (>6 days)	Label	Subjects (%) with the same spatio-spectral tendency
		*x*	*y*	*z*					
Valence	Frontal central	0	52	6	10	Beta	40	Negative	30
						Gamma	40		30
	Central midline	3	1	49	6	Beta	40	Negative	40
						Delta	30		30
	Right occipital	20	−56	−6	19	Beta	40	Positive	30
Arousal	Superior parietal	3	−42	58	5	Beta	25	Low	25

*The percentage of subjects with the same relatively day-stationary spatio-spectral oscillations correlated with the binary states and with the same tendency towards one of the binary states are identified*.

## Discussion

This work explored the extent of intra- and inter-individual EEG non-stationarity associated with emotional responses using the data-driven approach of an ICA. For the analysis of the 8-day EEG sessions of 10 subjects, an ICA-based analytical framework was conceived to identify the neurophysiologically interpretable spatio-spectral source oscillations for each single-day session, exploit their statistical link to the dichotomized emotional states, and assess the (non)stationary emotion-related EEG patterns along days and their inter-subject commonality. Results indicated substantial salient EEG non-stationarity in the numbers and locations of brain sources of interest as well as their spectral modulations to the emotional responses. However, this work did not attempt to disentangle the underlying neural mechanisms driving such vivid non-stationarity; rather, it sought to empirically demonstrate how clearly they emerge through source-level analysis. Leveraging neuroscience and machine-learning approaches, previous studies have yielded many important insights regarding affective computing, yet they mostly focused on single-day analysis given a group of subjects. This work substantially advanced the previous work by addressing the EEG non-stationarity in an ecologically valid multiday scenario that is considered to be a great challenge to the development of a robust, accurate, and generalized aBCI model for realistic applications.

### Integrity of the ICA for Exploring (Non)stationary Sources

In this work, we used ICA to parse scalp channel data into spatially fixed and temporally independent sources and evaluate their association with emotional responses. Unlike channel-level analysis which may be compromised by volume conduction (Jung et al., 2000; Onton and Makeig, 2006), the ICA algorithm theoretically isolates cortical and non-cortical source signals, such as muscle tension and eye movements, from the spontaneous signal mixtures recorded from the scalp sensors. Once the respective best-fitting equivalent dipoles of the derived ICs have been localized, the source-level outcomes enable a better understanding of brain source-specific neural oscillations and their behavior over time and across individuals. Nevertheless, the number of resolved ICs is the same as the number of sensors used to record the signal mixtures. Using a limited number of sensors is not likely to fully reflect the underlying sources, which could be unlimited (Onton and Makeig, 2006). In addition, among the decomposed sources, only a few of them, with homogeneous scalp maps, within-brain dipoles, and meaningful spectral oscillations, explain the relatively large data variance of the signals, potentially leading to more neurophysiologically accessible associations. The remaining ones, which either have stereotypical artifacts or low-energy non-dipolar scalp maps, are less relevant and can be omitted. The analytical rationale of an ICA has been successfully demonstrated for the analysis of various phenomena using different numbers of scalp channels (e.g., 32–250), such as motor imagery (Wang et al., 2012), motion sickness (Chuang et al., 2012), music appreciation (Cong et al., 2013; Lin et al., 2014), walking locomotion (Wagner et al., 2016; Artoni et al., 2017), stress level (Schlink et al., 2017), and affective state (Onton and Makeig, 2009; Rogenmoser et al., 2016; Banaei et al., 2017). Comparing previously reported IC outcomes in terms of the number of cortical ICs vs. the number of channels, e.g., 5–15 ICs with 31 channels (Onton and Makeig, 2006), 8–15 ICs (mean: 11.2) with 32 channels (Wang et al., 2012), 12–29 ICs (mean: 20.3) with 128 channels (Banaei et al., 2017), 15–25 (mean: 18.4) with 248 channels (Gramann et al., 2010), and 9–31 ICs (mean: 16.0) with 250 channels (Onton and Makeig, 2009), this work, yielding an average of 11.20 ± 1.96 interpretable cortical ICs from 30-channel EEG signals across 80 single sessions, was deemed acceptable. Moreover, the meaningful brain ICs derived from this work were all located in frontal, central, sensorimotor, parietal, and occipital regions of the cortex, consistent with previous findings (Onton and Makeig, 2006; Chen et al., 2010; Lin et al., 2014; Rogenmoser et al., 2016; Wagner et al., 2016; Banaei et al., 2017; Schlink et al., 2017), regardless of the channel set up used. No ICs located in deeper sub-cortical regions were resolved. This may be attributed to the fact that scalp EEG signals are less sensitive to neural activation stemming from deep subcortical structures. This work therefore cannot draw any conclusions on whether deeper structurers behave more (non)stationarily compared to the explored cortical ICs, especially for limbic and paralimbic areas involved in emotion processing revealed by other neuroimaging modalities, such as functional magnetic resonance imaging (fMRI) and positron emission tomography (PET; Blood et al., 1999; Phan et al., 2002; Trost et al., 2015). Accordingly, this issue needs to be taken into account during the interpretation of the stationary EEG sources in the present work.

From the 8-day EEG signals of 10 subjects, each subject returned an 8-day average of 88.50 ± 16.28 cortical ICs, from which was assessed which ICs were relatively reproducible across the 8 days. Nine aggregated cortical IC clusters located in frontal (left, right, and central), central midline, sensorimotor (left and right), superior parietal, and occipital (left and right) regions showed an average 8-day reproducibility of 76.03 ± 10.40 (min: 60.89 ± 28.36, max: 88.57 ± 10.98%) over 10 subjects (mean dipolarity: 95.99 ± 1.04%, see Figure 5). In other words, the nine identified ICs appeared at least, on average, for four (50%) and six (75%) of the 8 days. In an attempt to further quantify inter-subject commonality across the 10 subjects (as reflected in an IC present over 6 days, i.e., with a reproducibility >75%), 5 to 10 subjects possessed the same distinctive relatively day-stationary sources with a mean inter-subject commonality of 71.11 ± 18.33%. In all subjects, the frontal central source could be repeatedly seen on at least 6 days. If the criterion for cross-day reproducibility became more stringent (not presented in Results), the range and mean of inter-subject commonality considerably decreased towards the value reached at 8 days (7 days: 20%–90% (mean: 54.44 ± 23.51%), 8 days: 0%–70% (33.33 ± 22.91%). Among these cross-day criteria, the central midline source, rather than the frontal central source, was found relatively stationary across days and individuals (6 days: 90%, 7 days: 90%, and 8 days: 70% vs. 6 days: 100%, 7 days: 70%, and 8 days: 40%, respectively). Such varied cluster-to-cluster reproducibility indicated that the cortical EEG sources of interest behaved distinctly across multiple days, precisely considered to reflect intra-individual non-stationarity. The absence of the ICs on certain single-day sessions may be in part due to the source origins whose projected signals were neither strong nor distinct enough to be detected at the scalp and subsequently resolved by ICA (Onton et al., 2006). Previous EEG-ICA studies mostly assessed task-related spatio-spectral EEG oscillations by summarizing single-day analyses from a group of subjects (Onton and Makeig, 2006; Chen et al., 2010; Gramann et al., 2010; Chuang et al., 2012; Wang et al., 2012; Lin et al., 2014; Rogenmoser et al., 2016; Wagner et al., 2016; Banaei et al., 2017; Schlink et al., 2017). Less effort was invested in the issue of intra-individual differences across EEG sources. We believe that the qualitative IC outcomes from the 8-day sessions in this work have provided an opportunity to better understand EEG non-stationarity.

### Intra- and Inter-individual Differences in Spatio-spectral Correlates of Emotional Responses

Even though the nine cortical IC clusters were significantly compromised by EEG non-stationarity, some remained relatively consistent across days and individuals in response to the dichotomized emotional states (see Figure 6 and Table 1), especially in the valence category. Four of 10 subjects possessed the central midline source (BA 6, premotor cortex) on 6 of the 8 days, accompanying the beta suppression with the negative valence. Other outcomes included negative valence-induced beta and gamma suppression over the frontal central region (BA 10, anterior prefrontal cortex), negative valence-induced delta suppression over the central midline region, and positive valence-induced beta suppression over the right occipital region (BA 19, visual cortex), as derived from three subjects. In contrast, only two of eight subjects (2 of 10 subjects were excluded due to highly imbalanced labels) showed beta suppression in the superior parietal region (BA 5, superior parietal lobule) in low arousal. To the best of our knowledge, this work represents the first attempt to extract information from spatio-spectral EEG oscillations of emotional responses in the context of a longitudinal experiment (i.e., in the form of 8-day music-listening recordings interspaced over 2 months, roughly once per week). Due to a lack of direct evidence, the obtained outcomes were related to previous single-day work in terms of the localized brain regions and spectral oscillations. A meta-analysis study (Phan et al., 2002) that aggregated the findings of emotional activation from 55 PET and fMRI studies summarized the role of medial prefrontal cortex in emotional processing (reported by at least 40% of the included studies), which may support our findings on frontal central ICs. Further, it is plausible that emotional states reached during exposure to consonant music stimulate the additional drive of the motor system (Sammler et al., 2007; Lin et al., 2014). Several neurophysiological studies have also found that some music-modulated brain activity intervenes in emotion processing (Blood et al., 1999; Khalfa et al., 2005). Similarly, our results demonstrated informative IC sources located around the premotor cortex. Posterior (parietal and occipital) regions have been reported to be associated with emotional affect and intensity (Heller, 1993; Schmithorst, 2005), which may explain the engagement of parietal and occipital sources in this work. Engagement of multiple brain sources was ecologically true since music-induced emotion was accompanied by a rich involvement of reward, memory, self-reflective, and sensorimotor processes and engaged distributed brain networks across both cortical and subcortical regions (Trost et al., 2015). In contrast, with regards to the distinguishable spectral oscillations, most of them were seen in the beta band (frontal central, central midline, superior parietal, and right occipital regions), with fewer in the delta (central midline region) or gamma (frontal central region) bands. Previous EEG findings may provide partial support to these findings, notably those related to prefrontal beta and gamma asymmetry in valence (Daly et al., 2014), parietal beta asymmetry for motivation and emotion (Schutter et al., 2001), and widespread delta synchronization for music processing (Bhattacharya and Petsche, 2005). However, this work did not replicate certain representative spectral outcomes, such as fronto-midline theta enhancement for the positive valence (Sammler et al., 2007; Lin et al., 2010a) or frontal alpha asymmetry for valence distinction (Schmidt and Trainor, 2001; Davidson, 2004). Frontal central theta was sparsely observed in two subjects (yet did not result in an augmentation in positive valence), while frontal alpha modulation was only seen in the representative subject (see Figures 3, 4). This may be partly attributed to inter-day variability due to changes in mental states over the course of a multiday recording, such as in the form of mental fatigue modulating the lower frequency power of delta, theta, and alpha bands (Lal and Craig, 2002).

With regards to the within- vs. cross-day analysis of the representative subject (see Figure 3), it may be that the emotion-discriminative sources analyzed in the cross-day analysis could have been absent, and their spectral associations to the binary states could have behaved either reciprocally or even indiscriminately on certain days. Such day-to-day spectral variability was similar to findings using peripheral bio-signals (Picard et al., 2001) and in other EEG-related topics (Christensen et al., 2012). The underlying mechanisms of this cross-day discrepancy remain unclear based on this study’s outcomes, but could be partly attributed to the physiological modulation of behavioral and mental states, such as attention, stress, anxiety, and sleep quality. Previous EEG studies have reported that these factors indeed somehow modulate tasked-related EEG patterns. For example, neurophysiological correlates of mental fatigue differed between sleep-deprived and well-rested controls (Ahn et al., 2016), spectral oscillations fluctuated according to attentional demands (Wang et al., 2018), and acute stress affected the cognitive ability of brain-computer interface control (Garcia et al., 2019). It is reasonable to conclude that each of the aforementioned factors and their plausible interactions more or less concurrently confound the EEG patterns, leading to non-stationarity on different days. As such, exploring inter-subject commonalty for the relatively day-stationary sources is presumably more challenging. Given the criterion for IC’s cross-day reproducibility (>6 of 8 days), only a few subjects demonstrated same day-stationary ICs with a significant relationship to emotional responses (valence: 3–4 of 10 subjects, arousal: two of eight subjects). The inter-subject commonality more or less deteriorated if the same spectral tendency was involved (see Table 1). Previous neuroimaging studies have proven that individual differences associated with morphological differences in brain anatomy (e.g., gray and white matter volume), exhibiting a wide range of basic and higher cognitive functions (Kanai and Rees, 2011). The distinctive brain structures and functional patterns involved may serve as a useful source of information to study their links to human personality, behavior and cognition (Kanai and Rees, 2011; Liu et al., 2019). Particularly, personality is considered a dominant factor contributing to individual differences in emotion perception and experiences (Eysenck, 1998) and regulation strategies (Gross and John, 2003). These data could thus serve as a physiological indicator, for example, to correlate with stress resilience (Brouwer et al., 2015) and emotional states (Subramanian et al., 2018). In addition, recent work has demonstrated that the EEG variability was considerably larger across individuals than across repeated sessions. Such inter-subject variability may be increased while engaging in a more cognitive-oriented task (Melnik et al., 2017). In contrast, with regards to the ICA, the non-identical cortical and subcortical brain volumes likely make the size and/or orientation of EEG sources quite variable. Therefore, all individuals may not contribute the same ICs located in brain regions of interest (Onton and Makeig, 2006). Taken together with intra-individual variability, this low inter-subject commonality for the same tendency of day-stationary spatio-spectral correlates of psychophysiological emotional responses seems reasonable and realistic.

### Negative Impact of Nonstationary Spatio-spectral Oscillations to aBCI

This work has empirically demonstrated strong intrinsic intra- and inter-individual variability in emotional responses using source-level analysis. The numbers and locations of EEG sources of interest and their discriminative spectral profiles were found to be different across days and individuals. As such, EEG signals recorded from the scalp may be more substantially different from one another since they consist of linear mixtures projected from multiple non-stationary cortical sources (in addition to non-cortical artifactual sources). This source-to-channel projection may explain why the inter-day data clusters of the same emotion had more variability more than the inter-emotion data clusters within one day, as revealed by the channel-level analysis (Lin et al., 2015). Our exploratory findings also demonstrate why the day-independent (Chai et al., 2017; Lin et al., 2017; Liu et al., 2018) and subject-independent (Soleymani et al., 2012; Li et al., 2018, 2019) emotion prediction scenarios (i.e., a single generic model works on multiple days or on multiple subjects) were more challenging than their day-dependent and subject-dependent counterparts. Accordingly, this work highlights an urgent need to incorporate typical machine-learning frameworks with advanced signal processing [e.g., robust principal component analysis (Lin et al., 2017) stationary subspace analysis (Kaltenstadler et al., 2018)] and model calibrating steps (Chai et al., 2017; Liu et al., 2018; Li et al., 2019) to obviate the negative interference of discrepant EEG distributions across sessions obtained on different days or from different individuals. Furthermore, alternative to leveraging a unique model for the prediction of different days or individuals, future effort can be devoted to evaluate an ensemble learning framework (Chuang et al., 2014) that generates multiple classifiers to learn distinctive EEG distributions of emotional responses and strategically combines their multiple decisions. Thus, effectively monitoring/alleviating the EEG non-stationarity and adapting an existing model(s) accordingly will facilitate the translation of laboratory-oriented demonstrations to real-life aBCI applications.

## Conclusion

This work exploratorily demonstrated the extent of intra-individual and inter-individual EEG non-stationarity associated with emotional responses using the data-driven approach of an ICA. To this end, this work conducted an 8-day music-listening experiment (i.e., roughly interspaced over 2 months) and recorded whole-scalp 30-ch EEG data from a group of 10 subjects. Results from this large dataset (i.e., 80 sessions) indicated substantial EEG non-stationarity in the numbers and locations of brain sources of interest as well as their spectral modulations to emotional responses. Only less than half of subjects (two to four) demonstrated the same relatively distinct day-stationary (source reproducibility >6 days) spatio-spectral tendency towards one of the binary emotion states. Since previous works mostly focused on single-day/-session recordings and sensor-level analysis, this work substantially advances the work of these previous studies by exploiting EEG non-stationarity in an ecological multiday scenario. This is considered a great challenge to the development of a robust, accurate, and generalized aBCI model aimed at realistic applications.

## Data Availability Statement

The datasets for this manuscript are not publicly available at this moment because the data recording is ongoing for more subjects. Future requests to access the datasets should be directed to the corresponding author.

## Ethics Statement

The studies involving human participants were reviewed and approved by Human Research Protection Program of Kaohsiung Medical University, Taiwan. The patients/participants provided their written informed consent to participate in this study.

## Author Contributions

Y-WS conducted the experiments, analyzed the data, and wrote the corresponding parts of the article. Y-PL conceived and supervised the experiments and data analysis and wrote and revised the article.

## Conflict of Interest

The authors declare that the research was conducted in the absence of any commercial or financial relationships that could be construed as a potential conflict of interest.
